# “Becoming myself”: how participants in a longitudinal substance use disorder recovery study experienced receiving continuous feedback on their results

**DOI:** 10.1186/s13011-020-0254-x

**Published:** 2020-01-23

**Authors:** Thomas Solgaard Svendsen, Jone Bjornestad, Tale Ekeroth Slyngstad, James R. McKay, Aleksander Waagan Skaalevik, Marius Veseth, Christian Moltu, Sverre Nesvaag

**Affiliations:** 1 Centre for Alcohol and Drug Research, Stavanger University Hospital, Stavanger, Norway; 2 Department of Social Studies, Faculty of Social Sciences, University of Stavanger, Stavanger, Norway; 3 Department of Psychiatry, Perelman School of Medicine, University of Pennsylvania, Philadelphia, PA USA; 4 Philadelphia VA Medical Center, Philadelphia, PA USA; 5 Department of Clinical Psychology, University of Bergen, Bergen, Norway; 6 Department of Psychiatry, District General Hospital of Førde, Førde, Norway

**Keywords:** SUD, Longitudinal, Recovery, Feedback, Results, Research participation

## Abstract

**Background:**

Being a participant in longitudinal follow-up studies is not commonly a factor considered when investigating useful self-change aspects for individuals attempting recovery from substance use disorder (SUD). This study reports on how ongoing monitoring, and feedback on data results in a longitudinal follow-up study of SUD recovery were perceived by individuals who had achieved long-term abstinence and social recovery.

**Methods:**

Interviewers with first-hand experience with the topic conducted interviews with 30 participants and analysed the data using a thematic analytic approach within an interpretative–phenomenological framework.

**Results:**

Analyses resulted in the following themes. 1) Ongoing short text messaging (SMS) monitoring: helped participants by offering recovery milestones and reminders of the past. 2) Feedback on data results helped participants track physical and cognitive recovery: “I am more like myself”. 3) Using feedback in treatment: understanding the importance of a functional brain to participants may help with long-term retention in treatment.

**Conclusions:**

Self-changes that were challenging to detect on a day-to-day basis were available for reflection through longitudinal study participation, including ongoing monitoring and feedback on the results, allowing personal consolidation of change processes. Clinical services could benefit from continuing development and implementation of such technology for ongoing monitoring and feedback on assessments to motivate self-change in SUD recovery. The development of guidelines for providing the results of research assessments to individuals could help reduce attrition in research projects and support recovery and healthy choices for study participants.

## Background

Most professionally delivered substance use disorder (SUD) treatments target the more acute, early recovery needs [1], although recovery from substance use disorder commonly requires long-term efforts and includes episodes of relapse and several treatment sequences using various treatment programs [2–7]. Longer term, positive personal changes that occur outside such formal treatments are often described as self-change processes [8] or natural recovery [9].

Continuing care management (CCM) programs have been developed to address challenges to long-term change, including ongoing monitoring, personalized feedback and reducing the time from relapse to treatment re-entry [10–12]. As an example of a CCM study (2 years duration), Scott, Dennis, and Foss [13] designed a Recovery Management Check-up model (RMC) with 448 participants randomly assigned to either quarterly assessments only, or assessments including the RMC. The goal was to study early detection and linkage to treatment for relapsing individuals. Compared with assessment-only participants, people assigned to RMC were significantly more likely to return to treatment sooner during periods of relapse, and to stay in treatment longer than controls. The study by Scott et al. [13] and similar ones [11, 14] clearly show that there does not exist a “one size fits all” method in terms of the amount of time that is necessary to obtain recovery from SUD. Such an approach is critical, because the definition of what long-term intervention processes entail in SUD research studies is not fixed. A meta-analytical review on CCM by Blodgett et al. [15] showed that only eight of the 33 reviewed studies retained patients for 12 months or longer, suggesting that only shorter-term effects of interventions are measured in most studies on CCM.

This line of thinking is mirrored in the focus of recent standardized treatment packages on continuous monitoring and feedback of outcomes during active treatment. Clinical feedback from patients to clinicians during treatment is shown to enhance collaboration and improve outcomes [16], especially for patients who deteriorate [17, 18] or who are not on track to recovery [19].

Evidence suggests that one of the working mechanisms of clinical feedback is through preventing drop-out [20]. One key problem with such treatment-supporting systems is to determine what happens when treatment ends, and how long-term consolidation and personal change processes continue.

Although ongoing monitoring and personalized feedback are perceived as important in CCM, it is generally not common to provide feedback from individual research data to research participants. However, some participants express a wish to receive such information [21–23]. Research on discussions regarding the dissemination of results at aggregated levels [24] and procedural guidelines for reporting individual research results are scarce [25]. Barriers to discussions in these studies revolved around ethical aspects of disseminating sensitive health information, staff resources, and researchers’ attitudes toward providing results. Discussions on how the provision of results could benefit study participants in terms of access to gained knowledge, and as a way for research studies to “give back” in return for study participation, have also been reported [24–26].

This exploratory study is part of an ongoing longitudinal, 10-year clinical cohort follow-up study investigating long-term courses and outcomes in a recruited sample of individuals with SUD [27–30]. Our participants formed a subsample of 30 individuals who met strict criteria of long-term substance abstinence and social recovery [31, 32]. The main aim of the study was to investigate the processes perceived by participants to achieve such outcomes. In this, we investigated how long-term and ongoing monitoring, and personalized feedback on results were perceived by the informants, and whether participation promoted self-change processes.

## Methods

We used a thematic analytic approach [33, 34] developed within an interpretative–phenomenological framework [35]. The interpretive approach meant that study data were generated both from a reflexive dialogue between participants and researchers, as well as from a member of the study team checking procedures throughout the interviews. The phenomenological element in our approach entailed the collection of significant knowledge from individuals with lived experience of SUD to discover and interpret the meaning of such experiences [36]. We developed objectives and procedures within a user-involved research framework [37, 38]. We recruited two service users with first-hand knowledge of long-term recovery from SUD (T.E.S. and A.W.S.). They contributed in developing the interview guide, conducting the interviews, analysing the data, and reporting the study. These collaborative aspects and service user involvement in this study are detailed further in Veseth et al. [32].

### Sample and recruitment

The sample was recruited from the ongoing Stayer study (*n* = 202), a 10-year, prospective, naturalistic follow-up study of change trajectories following SUD in Rogaland, Norway. Participants were included between March 2012 and December 2015 from outpatient and residential treatment facilities at the start of treatment. Inclusion criteria included persons starting a new treatment sequence who fulfilled the criteria for SUD and were aged ≥16 years. Retention rates in the study were 89% at the 12-month assessment and 75% at the 72-month assessment. We recruited sub-study participants for interview consecutively at their 4- or 5-year follow-ups. The Stayer study team conducted a screening process based on objective criteria for stable substance abstinence and social recovery (see Measures). 34 eligible candidates were contacted; of these, three individuals refused participation and one was unreachable. Sample size was decided based on the stability of findings, reviewed after recruiting 19 and 26 participants [39]. We stopped recruiting after 30 participants because we considered the last four interviews (i.e., numbers 27–30) *not* to contribute substantially new information.

### The feedback context

We used bi-weekly short text messaging (SMS) tracking to gather data on the consumption levels of participants and their contact with treatment services. The decision to use bi-weekly monitoring was based on finding the optimal balance between gathering real-time data and not overburdening participants by using a protocol that was too demanding. The following SMS message was sent bi-weekly to the participants: “Hello. Please answer this SMS with a number ranging from 0 to 5 and yes or no,” where the number referred to recent substance use (ranging from 0 indicating no use to 5 indicating a subjective sense of considerable use) and the yes/no question referred to whether the participant had been receiving outpatient or residential treatment during the past 2 weeks. This bi-weekly SMS monitoring focused on the subjective experience of the participants as regards their bi-weekly consumption level, and not on counting the exact number of units or dosages injected or consumed. The SMS contact was always answered with a “thank you” message, regardless of the answers provided.

The baseline assessment in the study used 16 instruments and self-report forms; the quarterly assessments used eight instruments and self-report forms; and the 12-month assessment used 14 instruments and self-report forms (see Appendix).

Feedback on data results was provided if the individual participant expressed an interest in it: for example, if a participant requested feedback on Conner’s Continuous Performance Test II Version 5 but did not request feedback on other instruments or self-report forms. Thus, the feedback provided on data results varied both in the full cohort and in the subsample presented here.

### Measures

We used the following instruments in this study: The Drug Use Disorders Identification Test (DUDIT-C) to assess drug use [40]; the Alcohol Use Disorders Identification Test (AUDIT-C) to assess alcohol consumption [41]; the Symptom Checklist 90 Revised (SCL-90-R) to assess psychological functioning [42] based on the summarized Global Severity Index (GSI); the Behavior Rating Inventory of Executive Functions – Adult Version (BRIEF-A) to assess executive functioning [43]; and the Satisfaction With Life Scale (SWLS) to assess quality of life [44]. Substance abstinence was defined as a DUDIT-C score of 0 and AUDIT-C scores < 2. Relapse was defined as above the cut-off scores for either alcohol or drug use during the past 2 years. Social functioning was defined using four variables related to social functioning status: housing, income, friends without addiction, and participation in work or school. Participants who met all four social variables were categorized as having adequate social functioning. Here, recovery was defined as meeting criteria for both stable substance abstinence and adequate social functioning in the past 2 years.

### Interviews

Interviews were conducted between October 2017 and April 2018. We developed a semi-structured interview guide in line with the recommendations of Miles et al. [45], based on existing reports of factors facilitating SUD recovery, e.g., [7, 11, 46, 47], in collaboration with T.E.S. and A.W.S. The following focus areas guided the interview: (1) person-specific factors; (2) environmental factors; (3) treatment-related factors; and (4) experiences of participation in the Stayer study. Each theme was introduced with an open-ended question such as: “How would you describe your experiences with being a participant in the Stayer study?” We used follow-up questions that encouraged participants to relate their experiences to relevant contexts: for example, asking “Could you elaborate on what it was like to answer bi-weekly SMSs over several years?” or “Could you tell me more about what feedback from the study you have received?” To capture topics not adequately covered by the interview, participants were invited at the end of each session to provide any relevant information that had not yet been elicited.

Pilot interviews were conducted with two clinically recovered service users. All interviews were conducted by T.E.S. and A.W.S., who received training in semi-structured interviewing by J.B. The interviews provided us with a dataset that was developed through conversations between peers [48], because the way questions are asked is necessarily impacted by the answers participants provide [32, 49]. Interviews (mean duration 57 min; range 27–96) were conducted at Stavanger University Hospital (*n* = 25), at the participant’s home (*n* = 1), and by telephone (*n* = 4). Interviews were audio recorded and transcribed verbatim for the purposes of analysis.

### Analysis

In our analysis, we employed a seven-step procedure of condensing meaning [34] outlined in Table 1. To strengthen the credibility of the study, four of the researchers (J.B., T.S.S., M.V. and C.M.) conducted the seven-step procedure independently. During collaborative meetings, the same researchers compared their interpretations, agreed on themes with accompanying quotes, and validated the findings by consensus [50], dedicating special attention to steps four to seven presented in Table 1. T.E.S. and A.W.S. were selected as critical auditors to review and provide detailed feedback during the analysis and writing process. In accordance with Hill [51], the critical auditors’ roles were to ensure the structural validity of findings and to validate that the themes successfully represented any important material, as well as ensuring that the wording captured the essence of the material. Both auditors received basic textual analysis training and participated in several collaborative analysis meetings. Auditors’ comments were written down and integrated as part of the final analysis.

**Table 1:** Steps of text condensation

1.Becoming familiar with the data through thorough reading of the transcribed interviews, forming a main impression of the experiences of the participants, and identification of potential important themes. A theme was defined as a verbalization capturing an important element of the data in relation to the research question, representing a patterned response in the dataset.	
2.Generating initial codes, which were defined as the most basic segments of the raw data that could be assessed in a meaningful way regarding the phenomenon.	
3.Searching for and developing candidate themes and sub-themes. Remaining codes were set aside in this phase in a separate category for the purpose of being further analysed and incorporated when appropriate.	
4.Reviewing themes to develop a coherent thematic map and considering the validity of individual themes in relation to the dataset.	
5.Defining and naming themes. Further refining and defining themes, identifying the essence of themes, identifying sub-themes and summarizing the contents of the main themes into what each researcher considered to best represent participants’ experiences. When our refinements no longer added substantially to the themes, the analytic process was ended.	
6.To determine the relevance of any theme we counted both the frequency of the relevant meaning units combined with our interpretation of how central the theme was perceived to be for the recovery process.	
7.Last, the tentative model of findings, with illustrative quotes, was sent to two fully recovered service users who served as critical auditors assessing the interpretations made through our descriptions of the central organizing concepts.	

### Ethics

The Regional Ethics Committee in Norway (201/1877) approved the study. Ethical issues were discussed throughout the research process, from planning process to publication. We obtained written informed consent from all participants prior to the study, and we took care in the interviews and in working with the material to treat participants’ experiences with respect [32].

## Results

Demographic, clinical, treatment, psychological, and social variables are displayed in Table 2. In presenting the results, we refer to 20–30 participants as ‘most’, 10–19 as ‘many’, and 5–9 as ‘some’ of the participants [50]. Participants described how long-term study participation with feedback on data results and ongoing SMS monitoring provided mechanisms to illuminate cognitive and psychosocial status and self-change in long-term SUD recovery processes. Information of a) ongoing monitoring, b) feedback on results, and c) participants’ reflections on using feedback in treatment comprise three sub-themes in the Results section.

**Table 2:** Baseline and follow up demographic, clinical, treatment-related, psychological and social variables

	Baseline (*n* = 30)	Year 1 (*n* = 30)	Year 2 (*n* = 30)	Year 3 (*n* = 30)	Endpoint assessment	
					Year 4 (*n* = 10)	Year 5 (*n* = 20)
*Demographics*						
Age, years	25.9 (5.5)					
Male/female, *n*	17/13					
Education, years	12.8 (1.8)					
*Substance use history*						
Age at initial use (years)	13.1 (1.8)					
Years of drug use	12.9 (6.0)					
AUDIT score	11.9 (11.4)	3.4 (7.6)	2.3 (4.1)	2.9 (6.8)	4.4 (7.0)	2.2 (3.2)
DUDIT score	29.0 (15.9)	6.6 (13.1)	3.1 (11.5)	1.9 (8.5)	0 (−)	0 (−)
*Treatment*						
Previous treatment attempts	1.3 (2.0)	–	–	–	–	–
Currently outpatient, *n* (%)	13 (43)	17 (57)	8 (27)	5 (17)	2 (20)	2 (10)
Currently inpatient, *n* (%)	17 (57)	5 (17)	4 (13)	2 (7)	0 (0)	0 (0)
Currently in self-help group^a^, *n* (%)	13 (43)	13 (43)	15 (50)	10 (33)	4 (40)	3 (14)
*Social variables*^*b*^						
Permanent housing, *n* (%)	15 (50)	25 (83)	25 (83)	26 (87)	10 (100)	21 (100)
Stable income, *n* (%)	16 (53)	21 (70)	27 (90)	27 (90)	10 (100)	21 (100)
Employed/student, *n* (%)	5 (17)	7 (23)	14 (47)	19 (63)	10 (100)	21 (100)
Abstinent friends^c^, *n* (%)	24 (80)	25 (83)	26 (87)	27 (90)	10 (100)	21 (100)
*Psychological measures*						
SCL90-R GSI	1.2 (0.7)	0.7 (0.7)	0.6 (0.5)	0.5 (0.4)	0.5 (0.4)	0.4 (0.5)
BRIEF-A GEC	67.2 (11.3)	57.2 (11.3)	54.9 (12.6)	51. (10.9)	52.5 (10.5)	50.4 (11.2)
SWLS, sum score	17.5 (6.8)	24.8 (6.7)	24.8 (5.2)	25.2 (5.4)	25.3 (2.7)	27.4 (5.0)

All numbers are the mean (± standard deviation), unless otherwise specified. Abbreviations: SCL-90-R GSI, Symptom Checklist 90 Revised Global Severity Index T-score; BRIEF-A GEC, Behavioural Rating Inventory of Executive Function Adult Version Global Executive Composite T-score; SWLS, Satisfaction With Life Scale; AUDIT, Alcohol Use Disorders Identification Test; DUDIT, Drug Use Disorder Identification Test

^a^Currently in self-help group, such as NA/AA and the like

^b^Social variables are positive responses to yes/no questions

^c^Friends without a history of substance use

### Thematic analysis

#### Ongoing SMS monitoring: recovery milestones and reminders of the past

Participants’ early efforts to cease substance use during the study period were heterogeneous and non-linear, with slips and relapses, and with variations in physical and psychological distress. Many participants described these efforts as a day-to-day struggle, with uncertainty as to whether they were going to be able to maintain abstinence from substances over time. Many participants used the SMS monitoring system to reflect on their current substance use, shifts in use patterns, and substance use treatment needs during these early effort phases.

I received some SMSs in these periods. I think it was like two or three, and then I thought: “I am not in treatment. Should I be in treatment because of how I am feeling now?” It is about receiving those SMSs in different periods of your life.

Many participants described that in the initial, challenging phases of abstinence, the SMS system functioned as a bi-weekly recovery milestone. A positive effect was attributed both to their personal feeling of coping and achievement, and to reporting on these achievements to an external person. After several years, some participants perceived that the SMS monitoring transformed from a motivational abstinence tool to more of a non-demanding routine task. Life without using substances had become the new “normal”, and intense efforts such as in the early effort phases were no longer needed to maintain abstinence.

In the beginning, I remember it was great to report that I had not been using substances. Sure. But now it is more automated. Like: “zero and no”. These days it has become more a part of my daily life. It has been so for many years.

Although staying abstinent was no longer described as being demanding to the same degree as in the early recovery phases, after several years without using substances, many participants still experienced the SMS as a useful tool to reflect on the desired status quo that they had achieved through their long-term efforts.

You get a reminder, and then you become aware that: “Ok, now I’m here or there”, right? Because time goes, and after a while you do not think as much about these things as you did when you stopped using. So, it is satisfying to think that: “Ok, I am still alright”, sort of. A reminder about that.

### Feedback on data results: “I am more like myself”

Most participants were worried that prolonged substance use had reduced their cognitive and psychosocial capabilities permanently and were interested in receiving information on their cognitive and psychosocial status. Such an interest in information was evident for many participants, both when using substances and during periods of remission. Participants reported that the information provided on cognitive functions—such as memory capacity—that improved during periods of abstinence from substance use was particularly motivating in their efforts to sustain abstinence.

It has been great! I have received feedback that it has improved in some periods and worsened in others. But the periods when I can actually physically feel that my memory is back, I am more like myself. It has been fucking great.

As participants continued in the study over time, periods of using substances, and of abstinence, provided opportunities to learn from different phases in their recoveries. Many participants described that the comparison of experiences of abstinence and use motivated them to continue abstinence, because progress and development were visualized through this comparison. Attention and impulse control functions were highlighted as having especially motivational aspects when comparing past and current test results.

It has been super-cool to see. I have taken many of these tests when I used, and was intoxicated and everything. And from when I started to get a few months abstinent and meet (*the research assistant*), and he has told me about the differences between then and now, and my reaction functions and all this brain stuff. He has showed me results that there is no doubt that it is starting to re-connect … the system.

All participants had experienced several prior attempts at treatment for substance use before starting participation in the study and eventually gaining full recovery. Many participants described the importance of knowledge about the long-term efforts that are often needed to achieve recovery from SUD. Stopping using substances and engaging in treatment were described as important parts of these efforts, but a focus on the comprehensive, long-term changes and processes in daily life was underlined as equally important by many participants.

Because then you know that the brain is working faster, so to speak. And I also know that it takes time. Because that is what the study shows. And it does! It takes years! It is not all about quitting using, and then everything is ok the next day. It takes a long period of time. And that is why it is so hard, I think, it is a lifelong process, in varying degrees.

### Needing feedback in treatment: the importance of a functional brain

Both interest and worry, connected to cognitive and psychosocial status after prolonged substance use, were described by many participants. They described that treatment services in which they had been admitted had not used feedback on data results on cognitive and psychosocial functions, or ongoing monitoring as part of their interventions. Many participants described that gaining access to such information during treatment could have helped them.

I think for me, if I could get some cognitive feedback on my treatment progress: “You are not doing so well on these tests, your attention is a little off, your concentration too”. Right? For me, I think it is so important that my brain works.

Many participants reflected on the high attrition numbers from both outpatient and residential treatments for SUD. They described how visualization of progress could be used to a greater degree in general treatment services to motivate higher retention rates in treatment, and to help individuals abstaining from substance use after completing a treatment sequence. Self-changes that were challenging to detect on a day-to-day basis became available for reflection through feedback on the results:

For those who can stop using in periods, they can see progression, even if they cannot feel it themselves. But that they can see it, black on white, I think that could help.

The focus on SUD as a lifelong disease, and several other aspects connected to sickness and social segregation, were described by some participants as a potential barrier to desired progress and development in treatment. More feedback was desired on information that could assist in showing help-seeking individuals that hope for change is possible.

I see that there are a lot of people in treatment who are mentally fucked. They are narrow minded. To get permission to use many of those study questions to open up a bit and see that: “Fuck, we can think too! We are not as stupid as we sometimes think!” It is because we are so fucking locked on those bad thoughts about ourselves. So, I actually think that these questions can be mentally good for you.

## Discussion

This study contributes to research on first person experiences of participation in a naturalistic longitudinal study on SUD. Our findings suggest that longitudinal study participation can promote self-change, similar to continuing care management programs for SUD [3, 13]. Feedback and ongoing SMS monitoring was perceived as relevant facilitators over a range of phases: active substance use; phases with substance use; phases of abstinence during treatment; and several years not dominated by substance use.

Calling attention to progress that could be challenging to detect on a day-to-day basis was often described as especially important during the first months after stopping using substances. In these periods, the participants were often involved in intensive outpatient or residential treatment efforts, and they reflected on how feedback could be incorporated in these settings to facilitate changes. This is in line with studies showing that clinical feedback from clinicians to patients improves outcomes [52].

Feedback and SMS monitoring functioned as reminders that focusing on long-term efforts and comprehensive self-changes are often necessary in recovery from SUD. One problem with treatment-supporting systems is what happens when treatment ends, with the long-term consolidation and personal change processes that are essential for sustained recovery still to be accomplished. Our findings suggest that longitudinal follow-up studies, using methods including ongoing monitoring and feedback on data results, can serve as facilitators for long-term consolidation during recovery from SUD.

### Implications for clinical service delivery

Our findings indicate that ongoing monitoring and feedback on the results were experienced as useful by participants during their recovery from SUD. We underline one of the arguments by Scott et al. [13] that monitoring individuals can be useful in self-change processes, and that receiving documentation of progress is an important aspect of SUD recovery. Following this line of thinking, McKay [46] argues that the most important aspects of being able to sustain long-term SUD recovery are reinforcements that make continued abstinence more appealing or rewarding.

### Implications for research

Dissemination of results has been done both at individual and aggregated levels in the Stayer study from its outset. This has provided the study with pragmatic organizational tools to “give back” information to participants, as one of several means to facilitate higher follow-up rates over time [30] and underlining the fact that the participant is more than a means to knowledge generation [25].

Researchers conducting longitudinal studies are uniquely placed to design studies that are meaningful for participants, directly linked to ethical and societal obligations to disseminate research results at individual and aggregated levels [24]. Discussions are necessary to make research data accessible for participants. These discussions should include the formats in which results should be communicated, whether the information could be potentially harmful for participants and, if so, how such challenges should be met.

Future health studies could benefit from including ongoing monitoring and feedback on results as embedded parts of the study design, because study participation per se can potentially promote self-change and contribute to high retention rates.

In this study, feedback on data from all study assessments was not given routinely but provided if—and when—the participant requested it. In designing the method of data dissemination in this manner, participants who did not desire feedback did not have to receive it. The study thus demonstrates a “less is more” approach to feedback and is an example that data feedback can be provided in pragmatic and non-demanding manners, depending on what information the study participants wish to receive.

### Limitations

Our findings are context-dependent for the participants and setting in which the study was conducted. Possible significant findings might have been excluded as a result of the rich level of data. The study participants were recruited from the same outpatient and residential treatment facilities in the region of Stavanger, Norway. This limitation could have affected the study results and their transferability to other contexts. A high percentage of participants had good functioning levels prior to experiencing SUD. Hence, this was a relatively homogeneous group of good prognosis patients, as would be expected when using social recovery as an inclusion criterion. However, this is not to say that these patients were not at risk of long-term functional disability. In addition, it does not compromise the credibility of the findings, even if it limits transferability to the most severe and prolonged SUD conditions.

## Conclusions

Feedback and ongoing monitoring can serve as useful elements in longitudinal follow-up studies on SUD recovery, contributing to the recovery processes for the participants, and to high retention rates in studies. Clinical services could benefit from implementing techniques using ongoing monitoring and feedback on assessments to motivate recovery.

## Data Availability

The dataset used in this study is 900 pages of transcribed data from 30 individual interviews. The data that support the findings of this study are available from Centre for Alcohol and Drug Research, Stavanger University Hospital, Stavanger, Norway, but restrictions apply to the availability of these data, which were used under license for the current study, and so are not publicly available. Data are however available from the authors upon reasonable request and with permission of Centre for Alcohol and Drug Research, Stavanger University Hospital, Stavanger, Norway,

## Appendix

### Measures of neurocognitive and psychosocial functioning

Regional quality register for treatment of addiction

Alcohol Use Disorder Identification Test (AUDIT)

Drug Use Disorder Identification Test (DUDIT)

The Symptom Checklist 90 Revised (SCL-90-R)

Snaith–Hamilton Pleasure Scale (SHAPS)

Satisfaction With Life Scale (SWLS)

Pittsburgh Sleep Quality Index (PSQI)

The Montreal Cognitive Assessment (MOCA)

Wechsler Abbreviated Scale of Intelligence (WASI)

Iowa Gambling Task (IGT)

Stroop

Behaviour Rating Inventory of Executive Function–Adult Version (BRIEF–A)

Trail Making TEST (TMT) Parts A and B

Conner’s Continuous Performance Test II Version 5 (u II V.5)

Adult ADHD Self-Report Scale (ASRS-v1.1)

Revised NEO Personality Inventory (NEO PI–R)

## Abbreviations

AUDIT-C: The Alcohol Use Disorders Identification Test
BRIEF-A: The Behavior Rating Inventory of Executive Functions – Adult Version
CCM: Continuing care management
DUDIT-C: The Drug Use Disorders Identification Test
RMC: Recovery Management Check-up
SCL-90-R: The Symptom Checklist 90 Revised
SMS: Short text messaging
SUD: Substance use disorder
SWLS: The Satisfaction With Life Scale (SWLS)
